# Reward Anticipation in Ventral Striatum and Individual Sensitivity to Reward: A Pilot Study of a Child-Friendly fMRI Task

**DOI:** 10.1371/journal.pone.0142413

**Published:** 2015-11-23

**Authors:** Branko M. van Hulst, Patrick de Zeeuw, Kellina Lupas, Dienke J. Bos, Sebastiaan F. W. Neggers, Sarah Durston

**Affiliations:** NICHE Lab, Department of Psychiatry, Brain Center Rudolf Magnus, University Medical Center Utrecht, Utrecht, The Netherlands; Indiana University, UNITED STATES

## Abstract

Reward processing has been implicated in developmental disorders. However, the classic task to probe reward anticipation, the monetary incentive delay task, has an abstract coding of reward and no storyline and may therefore be less appropriate for use with developmental populations. We modified the task to create a version appropriate for use with children. We investigated whether this child-friendly version could elicit ventral striatal activation during reward anticipation in typically developing children and young adolescents (aged 9.5–14.5). In addition, we tested whether our performance-based measure of reward sensitivity was associated with anticipatory activity in ventral striatum. Reward anticipation was related to activity in bilateral ventral striatum. Moreover, we found an association between individual reward sensitivity and activity in ventral striatum. We conclude that this task assesses ventral striatal activity in a child-friendly paradigm. The combination with a performance-based measure of reward sensitivity potentially makes the task a powerful tool for developmental imaging studies of reward processing.

## Introduction

Reward processing, and reward anticipation in particular, have been implicated in child and adolescent disorders such as attention-deficit/hyperactivity disorder (ADHD) [1], oppositional defiant disorder (ODD) [2] and depression [3]. Functional magnetic resonance imaging (fMRI) studies have addressed reward anticipation using the monetary incentive delay (MID) paradigm [4]. In this paradigm, a cue is presented at the beginning of each trial, signaling the amount of monetary reward that can be won (or lost) on the upcoming trial. By contrasting trials with different monetary cues, brain activation related to reward anticipation can be assessed [4]. In adults, the MID paradigm has been shown to reliably activate ventral striatum in anticipation of reward (e.g. [5,6]). Yet the symbols used in the task are highly abstract and there is no storyline. Accordingly, it has not often been used successfully with younger children and in particular those with developmental disorders.

We designed a child-friendly version of the task to be compatible with an fMRI environment [7]. We focused on making the task more engaging, as sustained attention is not yet fully developed at school age, and can be particularly challenging for children with developmental disorders. Furthermore, we used response times to develop a measure of performance that captures individual reward sensitivity, but does so in a manner that is independent of intra-individual variability in response times. This is important as intra-individual variability in response times is often increased in developmental disorders and can confound statistical inferences [7,8]. The inclusion of a performance measure of reward sensitivity is essential to the interpretation of changes in activity of ventral striatum and can aid the generalization of results to a real life construct of reward sensitivity. Other studies that successfully modified the MID task to be more suitable for use with children have reported on task performance, but have not considered the relationship between task performance and brain activation in the interpretation of their results [9,10].

In the MID task, faster reaction times signify better performance, as participants are instructed to press a button as fast as possible following a cue. Accordingly, the anticipation of greater reward may elicit faster reaction times [11]. Nevertheless, the effect of performance on brain activation has rarely been studied in the context of this task. This is likely related to the typical implementation of a staircase procedure in this task, designed to keep accuracy constant at a predetermined level (usually 60–70%) for all participants. Task performance then appears equal across participants. However, within individual participants, task performance (as expressed by mean reaction times, accuracy scores or both) may still differ across incentive conditions. Such a difference across incentive conditions may vary between participants and could therefore be operationalized as a behavioral measure of individual reward sensitivity. Following this rationale, we devised our measure of individual reward sensitivity by comparing rank-ordered reaction times across incentive conditions [7]. In previous studies, we have used our child-friendly version of the MID-task to show that participants with ADHD had lower behavioral sensitivity to reward compared to typically developing controls [7,8].

In this pilot-study, we set out to validate our child-friendly, modified MID task for use as a developmental fMRI paradigm. First, we tested whether it could elicit ventral striatal activation during the anticipation of reward in typically developing children and young adolescents. Second, we hypothesized that our individual measure of reward sensitivity should be associated with activity in ventral striatum. If so, future studies could use such a performance measure in the interpretation of their neuroimaging results.

## Methods

### Participants

A total of 18 right-handed children and young adolescents were recruited through schools in the wider Utrecht area. After screening for data quality (for details see S1 Text) two participants were excluded from further analyses due to anatomical abnormalities and three participants were excluded due to excessive motion during acquisition of the fMRI data. Data from six male and seven female participants, with an average age of 12.2 years (range: 9.5–14.5) and average IQ of 116, were available for fMRI analyses (see Table 1). The parents of participants reported no psychiatric diagnoses on the DISC-IV interview [12]. After the study procedures had been explained, all parents and adolescents (aged 12 and older) gave full written consent, while children provided verbal assent. The study and its procedures were approved by the medical ethical review board of the University Medical Center Utrecht (approval number: 08–081).

**Table 1:** Characteristics of the 13 subjects included in the fMRI analyses.

	Mean (SD)
**Age**	12.2 (1.62)
**Gender (m/f)**	6/7
**IQ**	116 (11.8)
**MRT 0ct**	424ms (63.2)
**MRT 5ct**	408ms (57.8)
**MRT 15ct**	405ms (63.7)
**B**_**0vs15ct**_	0.89 (0.31)

SD, standard deviation; m, male; f, female; MRT, mean reaction time, ms, milliseconds; ct, cents; B_0vs15ct_, shift in reaction time distribution between high reward and no reward trials.

### Monetary incentive delay paradigm

We used a modified version of the monetary incentive delay task [7] in a rapid event-related fMRI design. The task was made more engaging for use in children by adding a two-choice response selection aspect to the task. Trial sequence was as follows: first, the cue, a picture of a wallet with 0, 5 or 15 cents was shown (2000 ms), indicating the amount of money that could be won on the upcoming trial. Next, pictures of two popular cartoon figures (SpongeBob and Patrick Star from the SpongeBob TV-series) were shown (750 ms) and participants were to guess which cartoon figure was hiding the wallet by pressing either the left or the right response button. They were instructed to respond as fast as possible. Subsequently, a black screen was shown (500 ms) and finally, a thumbs-up or a thumbs-down picture, indicating a correct or incorrect choice, was shown (750 ms) along with the total amount of money won so far. The task was rigged in such a way that outcome of each trial was fixed; the choices made did not affect reward outcome. If participants did not respond within a 1250 milliseconds timeframe after the appearance of SpongeBob and Patrick Star, the feedback text “too late!” was presented. The task consisted of 240 four-second trials (80 trials per cue) divided evenly into four blocks and consequently took four minutes per block to complete. Each block had a fixed reward frequency of either 20% (low reward) or 80% (high reward). Participants were randomly presented with one out of two counterbalanced reward sequences (‘high-low-high-low’ or ‘low-high-low-high’), so that average reward frequency totaled 50% for the full task. For a schematic overview of task design see S1 Fig.

### Performance measures

The primary neuropsychological outcome measure was the shift in reaction time distribution between rewarded and unrewarded trials. This was quantified using linear regression of the rank ordered reaction times to high rewarded trials (15 cents) on the rank ordered reaction times to unrewarded trials (0 cents), as described previously [7]. We chose this measure as it is minimally influenced by differences in reaction-time variability, which is an important consideration in studying developmental disorders such as ADHD, where intra-individual variability in reaction times is greater than in typical development [13]. A regression coefficient smaller than one indicates faster responses time on rewarded then on unrewarded trials.

### fMRI acquisition

The study was run on a 3.0 T Achieva MRI scanner (Philips Medical Systems, Best, the Netherlands) using an eight-channel sensitivity-encoding (SENSE) parallel imaging head coil. For anatomical reference, a whole-brain three-dimensional fast field echo T1-weighted scan (200 slices; repetition time = 10 ms; echo time = 4.6 ms; flip angle = 8°; field of view, 240 x 240 x 160 mm; voxel size: 0.75 x 0.8 x 0.75 mm isotropic) was acquired. Whole-brain T2*-weighted echo planar images (EPI) with blood-oxygen level-dependent (BOLD) contrast (4 sessions; 126 volumes per session; 33 slices per volume; interleaved acquisition; TR = 2.00 s; TE = 35 ms; field of view = 240 × 240 × 116 mm; flip angle = 70°; voxel size = 3.0 × 3.0 × 3.5mm) oriented in a transverse plane were acquired. We acquired six dummy scans to allow for T1 equilibration effects.

### Preprocessing of fMRI data

FMRI data were analyzed using SPM8 (r4290) (http://www.fil.ion.ucl.ac.uk/spm/software/spm8) as implemented in Matlab 7.12 (Mathworks Inc., Natick, MA, USA). To correct for between-scan head motion, all images were realigned to the first volume using rigid body transformations, and subsequently resliced. Next, the data were temporally interpolated to the middle slice to adjust for time differences due to multislice image acquisition (i.e., slice time correction). The anatomical image was co-registered to the first fMRI image using the mutual information criteria method and subsequently normalized to Montreal Neurological Institute (MNI) space using unified segmentation, finally the image was resliced at a voxel size of 1.0 x 1.0 x 1.0mm. Functional images were normalized using the normalization parameters generated in this step, the images were resliced at a voxel size of 3.0 x 3.0 x 3.0mm. Finally, the fMRI images were spatially smoothed with a Gaussian kernel with a full width at half maximum (FWHM) of 8 mm. In addition, scan-to-scan movement was assessed using ArtRepair [14]. Scans with more than 0.5 mm scan-to-scan movement or more than 1.5% deviation from the average global signal, were replaced using a linear interpolation of the values of neighboring scans. Participants with more than 15% corrected scans were excluded from further analyses (for details, see S1 Text).

### Statistical analyses—performance

After testing for normality (Shapiro-Wilk test) and sphericity (Mauchly’s test), a repeated measures analysis of variance (ANOVA) was conducted to test for the effect of incentive magnitude on mean reaction time. Next, we tested for an effect of age on the shift in reaction time distribution using single-factor ANCOVA.

### Statistical analyses—fMRI

Statistical analyses were performed within the framework of the general linear model following a two-level procedure [15]. First level analysis involved the modeling of blood oxygenation level dependent (BOLD) activation invoked by 0, 5 and 15 cents cues as conditions of interest, and realignment parameters as potential confounders (condition of no interest) for each subject. Regressors were created by convolving delta functions coding for cue onset with a canonical hemodynamic response function (as implemented in SPM8) for each cueing category separately. The estimated regression coefficients for the 0, 5 and 15 cents condition were then contrasted resulting in three first-level contrast images for each subject: 0 versus 5, 0 versus 15 and 5 versus 15 cents. Data were high-pass filtered using discrete cosine basis functions with a 128 second cut-off. At the group level we conducted two analyses. First, we tested for brain activation related to the anticipation of reward by conducting one-sample t-tests on the contrast images of the three different contrasts separately. We corrected for multiple comparisons using a family wise error (FWE) correction on a predefined volume of interest (VOI) (i.e., small volume correction). This VOI was generated by combining the bilateral nucleus accumbens masks provided by the WFU PickAtlas toolbox [16] and dilating the resulting mask by one voxel in all dimensions (size of VOI: 151 voxels, 4077 mm^3^). Next, we tested for associations between reward-related brain activation and both task performance and age. We conducted six separate single-factor ANCOVAs, using the three first-level contrasts as dependent variables and the measure of task performance (the regression coefficient of rank ordered reaction times for the greatest contrast, 0 versus 15 cents; B_0vs15ct_), and age as separate continuous predictors (i.e., covariate of interest). Again we applied FWE correction for multiple comparisons restricted to the bilateral ventral striatum. When anticipatory ventral striatal activity was found, we additionally tested for an effect of reward frequency on brain activation by contrasting activity in blocks with high (80%) and low (20%) reward frequency (for the contrast in question). This was done using a one-sample t-test on a newly created contrast image, again we corrected for multiple comparisons using FWE in the same VOI.

## Results

### Task tolerability

18 Subjects participated in an fMRI session and performed the task without problem. Data for five subjects had to be excluded from the analyses due to anatomical abnormalities on the T1-weighted scan or motion during fMRI-scanning. They did not differ in terms of age, gender or IQ from the 13 subjects for whom fMRI data were available for analysis.

### Behavioral data

There was an effect of reward magnitude on mean reaction times (F(2,24) = 4.41,p = 0.023). Average reaction times were 424 ms, 408 ms and 405 ms across respectively no reward, low reward and high reward conditions. The mean B_0vs15ct_ (the shift in reaction time distribution between high reward and no reward) was 0.89 (SD 0.31), indicating faster responses on high reward then on no reward trials. There was no effect of age on the shift in reaction time distribution.

### fMRI data

**Fig 1:**
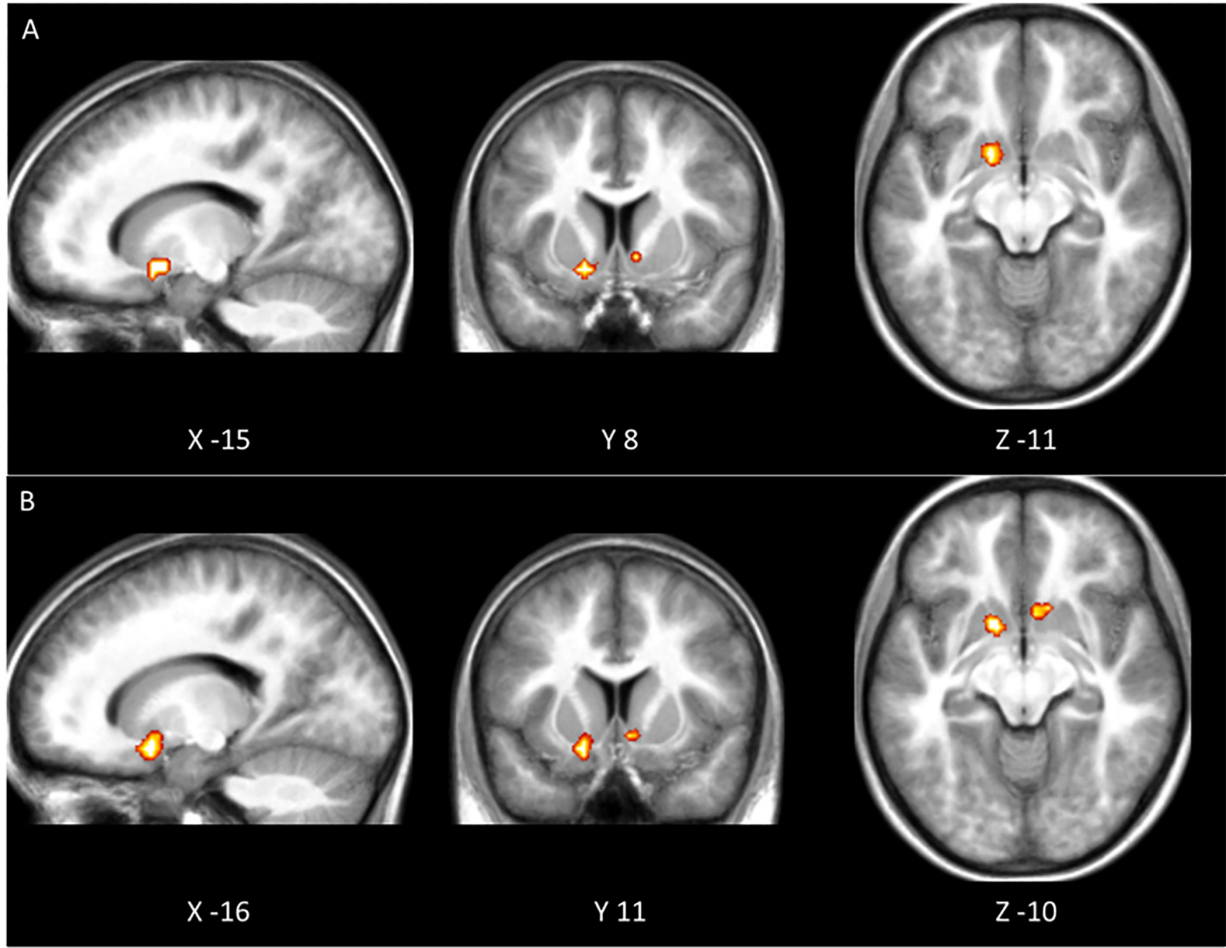
Ventral striatal activity is related to task performance. There was a positive correlation between activation in ventral striatum and the shift in reaction time distribution (B_0vs15ct_) (FWE corrected for multiple comparisons). Both panels display the T-map of a single-factor ANCOVA with B_0vs15ct_ as continuous predictor variable. Panel A shows the effect of task performance on the activation difference between low and high reward conditions. Panel B shows the effect of task performance on the activation difference between no reward and high reward conditions.

The first analysis aimed to test whether the MID task would elicit ventral striatal activation during reward anticipation. We contrasted the anticipation of 0 versus 5 cents, 5 versus 15 cents, and 0 versus 15 cents, separately. We found reward-related activation in the bilateral ventral striatum for the 0 versus 5 cents contrast (see Table 2), but not for the two other contrasts (5 versus 15 cents and 0 versus 15 cents). Next, we tested for a relationship between task performance and reward-related activity in ventral striatum by conducting a single-factor ANCOVA with the shift in reaction time distribution between high reward and no reward trials (B_0vs15ct_) as a continuous predictor. We found an association in bilateral ventral striatum for both the 5 versus 15 and the 0 versus 15 cents contrast (see Fig 1), but not for the 0 versus 5 cents contrast. We found no effect of reward frequency or age on anticipatory activity in ventral striatum.

**Table 2:** Activity in ventral striatum during the anticipation of reward.

	Contrast	L/R	P-value cluster	Cluster size	P-value peak	T-value peak	MNI coordinates
**Baseline activity**	0ct vs 5ct	L	0.016	7	0.041	4.29	-18, 5, -11
	0ct vs 5ct	R	0.021	5	0.024	4.69	15, 14, -8
Reward related activity	5ct vs 15ct	L	0.008	14	0.025	4.76	-15, 5, -8
	5ct vs 15ct	R	0.034	2	0.019	5.01	9, 5, -5
	0ct vs 15ct	L	0.003	25	0.019	4.90	-15, 5, -8
	0ct vs 15ct	R	0.017	7	0.006	5.91	6, 11, -8

L, left; R, right; MNI, Montreal Neurological Institute; ct, cents. All reported p-values were FWE corrected for multiple comparisons.

## Discussion

We set out to validate our child-friendly, modified MID paradigm as a task appropriate for use in developmental fMRI studies. The task successfully elicited activity in ventral striatum during the anticipation of reward. Furthermore, our task-based measure of reward sensitivity was associated with striatal activity during reward anticipation. The task was well tolerated by all subjects. In all, we conclude that this task has potential as a tool for assessing reward anticipation in developmental populations.

We found that the task was well tolerated by all subjects: children and young adolescents, ranging in age from nine to fourteen years, were able to comprehend and complete the modified MID-task in a scanner environment. In previous studies we have shown that children as young as six years old were able to comprehend and complete the task outside of the scanner [7,8].

The task successfully elicited activity in ventral striatum during the anticipation of reward. Furthermore, we found that a task-based measure of reward sensitivity correlated positively with striatal activation during reward anticipation. This suggests that anticipatory activity in striatum may be interpreted as a neurobiological measure of reward sensitivity in the context of this task. Such a direct interpretation of results is likely to be crucial in linking fMRI findings to real-world reward sensitivity, as assessed by differences in approach behavior. To date, results from studies reporting response-time differences across incentive conditions have been mixed [4,9,17–20] and when found, their relationship to brain activation has not previously been explored [17,19,20]. A study by Lamm and colleagues did report a relationship between task performance and brain activation in an MID paradigm, yet they only included performance as a nuisance variable [21]. In this way the potential value of task performance in the interpretation of fMRI results is left unexploited.

As in previous studies with adults [5,6], we found ventral striatal activation when contrasting no reward cues to low reward cues. However, we found no differences in activation when contrasting low reward cues to high reward cues. Stoppel and colleagues [22] have suggested that the effect of contrasting low reward to high reward is smaller than that between no reward and low reward, as the former contrast only probes reward magnitude and not reward itself. In addition, developmental studies using overlapping age-ranges (12–18 years) have reported reduced striatal engagement [17,21] and decreased discrimination between reward magnitudes [21]. As such, the difference between the anticipation of low and high reward may have been too subtle to elicit significant differences in brain activity, particularly given our relatively modest sample size. Likewise, the absence of an effect of age on anticipatory ventral striatal activity may be a result of limited power.

Furthermore, we only found an association between reward sensitivity and activity in ventral striatum for the contrast of high versus either low or no reward, but not for the contrast of low versus no reward. This may be related to increased inter-individual variability in striatal activation in high reward conditions, associated with individual differences in overall task performance. This increased variability may explain the lack of activation in the 0 versus 15 cents contrasts. Following this rationale, anticipatory striatal activation is more likely to be linked to a neuropsychological construct of reward sensitivity under high reward, than under low reward conditions.

To conclude, we show that a child-friendly version of the MID-task elicited activity in ventral striatum during the anticipation of reward in children and young adolescents. Furthermore, this striatal activation was related to inter-individual differences in a task-based measure of reward sensitivity. In all, this task assesses ventral striatal activity in a child-friendly manner. The combination with an individual task-based measure of reward sensitivity makes the task a potentially powerful tool for developmental imaging studies of reward processing.

### Limitations

First, the sample size of this pilot study was modest. This was in part caused by the exclusion of five datasets due to high subject motion or anatomical abnormalities (for details see S1 Text). As a result, any negative findings in this study should be interpreted with caution. In particular, tests to be expected to have a modest effect size (e.g. the manipulation of reward frequency) would be vulnerable to type II errors. Second, an important distinction between this version of the task and the classical MID is that participants had no control over their results (although they would be expected to have experienced control). This could have affected our results, as a recent study by Lorenz and colleagues [23] showed that subjective lack of control can attenuate ventral striatal activity. Third, it should be mentioned that the task was set up to have optimal statistical power with as many as 80 trials per condition. However, this led to a total time-on-task of 16 minutes, which may also have taxed sustained attention. Finally, the participants in our sample had above average IQ, necessitating prudence when interpolating findings to participants with an average IQ.

## Supporting Information

S1 Fig Task design. A schematic overview of task design is shown. Panel A shows the time course of a single trial. Panel B shows the two different reward frequency blocks. Panel C shows three different reward magnitudes that were used as trial types and contrasted for further analyses. Reprinted with permission from “Deficits in Cognitive Control, Timing and Reward Sensitivity Appear to be Dissociable in ADHD” by De Zeeuw et al, 2012, PLoS One. Jan;7(12):e51416.
(TIF)

S1 Text Screening of data quality. (DOCX)
