# Supplementary figures and images for: Reward Anticipation in Ventral Striatum and Individual Sensitivity to Reward: A Pilot Study of a Child-Friendly fMRI Task
Source: PLoS One. 2015 Nov 23;10(11):e0142413. doi: 10.1371/journal.pone.0142413 (PMC4657917; doi:10.1371/journal.pone.0142413)

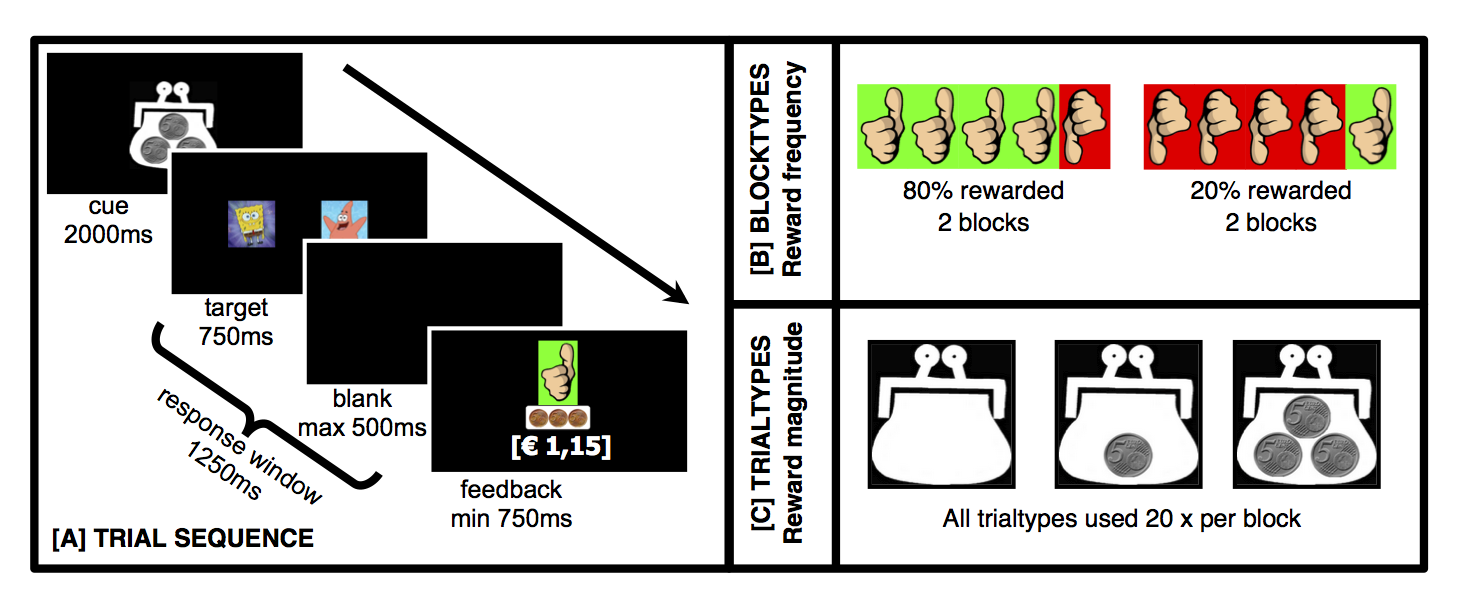

Supplement: S1 Fig — A schematic overview of task design is shown. Panel A shows the time course of a single trial. Panel B shows the two different reward frequency blocks. Panel C shows three different reward magnitudes that were used as trial types and contrasted for further analyses. Reprinted with permission from “Deficits in Cognitive Control, Timing and Reward Sensitivity Appear to be Dissociable in ADHD” by De Zeeuw et al, 2012, PLoS One. Jan;7(12):e51416. (TIF) [file pone.0142413.s001.TIF]
