# Supplementary material for: Reward Anticipation in Ventral Striatum and Individual Sensitivity to Reward: A Pilot Study of a Child-Friendly fMRI Task
Source: PLoS One. 2015 Nov 23;10(11):e0142413. doi: 10.1371/journal.pone.0142413 (PMC4657917; doi:10.1371/journal.pone.0142413)
Supplement: S1 Text — (DOCX) [file pone.0142413.s002.docx]

**Text S1. Screening of data quality.**

All T1-weighted scans were assessed by an expert radiologist. Following this step, two participants were excluded on the basis of anatomical abnormalities (one arachnoid cyst, one enlarged ventricular system). Scan-to-scan movement was assessed using ArtRepair (Mazaika, Hoeft, Glover, & Reiss, 2009). Scans with more than 0.5 mm scan-to-scan movement and scans with a more than 1.5% deviation from the average global signal, were replaced using a linear interpolation of the values of neighboring scans. Participants with more than 15% corrected scans were excluded from further analyses. One participant was excluded on the basis of this criterion. In addition, global quality was assessed with ArtRepair. Subjects were defined as outliers according to the 1.5 IQR criterion on the basis of their global mean (average contrast estimate across all voxels) and average residual error. Two subjects were excluded based on this criterion.
